# A Novel Biobjective Risk-Based Model for Stochastic Air Traffic Network Flow Optimization Problem

**DOI:** 10.1155/2015/742541

**Published:** 2015-06-09

**Authors:** Kaiquan Cai, Yaoguang Jia, Yanbo Zhu, Mingming Xiao

**Affiliations:** ^1^School of Electronics and Information Engineering, Beihang University, Beijing 100191, China; ^2^National Key Laboratory of CNS/ATM, Beijing 100191, China; ^3^Aviation Data Communication Corporation, Beijing 100191, China

## Abstract

Network-wide air traffic flow management (ATFM) is an effective way to alleviate demand-capacity imbalances globally and thereafter reduce airspace congestion and flight delays. The conventional ATFM models assume the capacities of airports or airspace sectors are all predetermined. However, the capacity uncertainties due to the dynamics of convective weather may make the deterministic ATFM measures impractical. This paper investigates the stochastic air traffic network flow optimization (SATNFO) problem, which is formulated as a weighted biobjective 0-1 integer programming model. In order to evaluate the effect of capacity uncertainties on ATFM, the operational risk is modeled via probabilistic risk assessment and introduced as an extra objective in SATNFO problem. Computation experiments using real-world air traffic network data associated with simulated weather data show that presented model has far less constraints compared to stochastic model with nonanticipative constraints, which means our proposed model reduces the computation complexity.

## 1. Introduction

With the rapid growth of air traffic, airspace congestion and flight delays are becoming serious due to demand-capacity imbalances in air traffic management (ATM) system, especially in convective weather conditions. Severe airspace congestion and flight delays not only deteriorate the service quality of airlines but also raise operational costs. According to statistics, in 2013 [1], the average punctuality rate of flights all over the world was only 80%. China endures an even lower rate, which was 73.1% for big airlines and 70.0% for small airlines. Among all factors that lead to flight delays, the impact of inappropriate flight plan and convective weather takes up a weight of 59.2%.

The network-wide air traffic flow management (ATFM), through constructing global predeparture flight plans (including departure time, flight routes, and arrival time at the waypoints or arrival airports) for all the flights planned to fly over the air traffic network, aims to balance the air traffic demand and ATM system capacity. In ATM domain, considered as one of the effective ways to alleviate airspace congestion and flight delays, ATFM becomes increasingly highlighted in the expanding air transportation system and has drawn a mass of attention to researchers. Single airport ground-holding problem was firstly proposed to minimize ground delays by optimizing departure time of a flight when its arrival airport capacity is insufficient [2]. Later on, the concerns evolved to multiairport ground-holding problem [3–5], air traffic flow management problem [6, 7], and air traffic flow management rerouting problem [8–12]. All above problems can be collectively labeled as air traffic network flow optimization (ATNFO) problem. An air traffic network [13–15] can be modeled as airports network, airspace sector network (nodes include airports and sectors), or air route network. ATNFO aims to minimize the cost of flight delays by optimizing flight plan over a given network and time horizon, taking into account the capacity limits of airports or airspace sectors.

The above ATNFO problems share a common nature; that is, the capacities of airports and airspace sectors are predetermined. However, due to the stochastic nature of the convective weather dynamics, it is hard to predict airport/airspace capacity accurately in future 24 hours (ATNFO is usually implemented more than 24 hours earlier to provide decision-making) in practice [16]. The convective weather dynamics often lead to capacity uncertainties for the airports or airspace sectors in a network. In the presence of capacity uncertainties, ATFM actions might be noneffective or impractical when adopting the deterministic ATNFO models. Hence, in recent years, the stochastic ATNFO (SATNFO) problem with uncertainties has aroused increasing concerns. Existing models for SATNFO problem can be classified into stochastic programming [17, 18] based methods and robust optimization [19, 20] based methods.

Deterministic models for ATNFO problem assume airports and airspace sectors capacity as constant values. However, stochastic programming based method considers the uncertainties of airports and sectors capacities by establishing probabilistic distributions for them and finds a solution that is feasible and optimal in some sense. The first attempt at stochastic programming in ATFM was made by Richetta and Odoni [21, 22]. They applied scenario tree [23, 24] to model the uncertainty of capacity and proposed a dynamic model for single airport ground-holding problem. Here, scenario tree is a kind of format to describe capacity change and its probability along time horizon. Later, the utilization of scenario tree was extended to stochastic model for multiairport ground-holding problem by Ball et al. [25]. In 2012, Agustín et al. [26] dealt with SATNFO problem under weather uncertainty by using stochastic model, which is an extension of their deterministic tight mixed 0-1 model [11]. In their work, a scenario tree which models the capacity uncertainties under storm scenarios is generated to represent the deterministic equivalent model for their stochastic mixed 0-1 program. Since scenarios in reality are numerous, especially when prolonging the time horizon (such as 24 hours) of ATFM, stochastic programming becomes intractable.

Robust optimization [27, 28] captures the probabilistic property of a problem by constructing appropriate limited uncertainty sets for uncertain parameters and then solves a solution that is feasible for all outcomes of the uncertainty sets. The solution is optimized in worst-case condition. Gupta and Bertsimas [29] and Bertsimas and Goyal [30] attempted to apply the idea of robust optimization to the capacity uncertainties in ATFM rerouting problem. Unfortunately, it is likely to get a very conservative solution and cause waste of airspace and time resource when using robustness optimization based method.

Both stochastic programming and robust optimization have the same basic idea for dealing with capacity uncertainties. Firstly, generate scenarios (scenario tree or uncertainty sets) to describe capacity uncertainties; then, optimize air traffic flow by using capacity scenarios as constraints. Their objective is to find a robust plan for all scheduled flights under uncertainty. But both methods have suffered computational intractability and given highly conservative solution.

In order to solve SATNFO problem efficiently, we propose a novel mathematic model that takes into account capacity uncertainties. In this model, we introduce the concept of operational risk to evaluate the effect of capacity uncertainties in ATFM and formulate it via probabilistic risk assessment methodology. A set of flight plans with lower operational risk is deemed to a more robust solution of SATNFO problem, which means the flight plans are more insusceptible to the capacity uncertainties of airports or airspace sectors. Hence, the SATNFO problem is formulated as a weighted biobjective 0-1 integer programming model with two objectives of minimizing the cost of total delays as well as the operational risks. The contributions of our novel SATNFO model are twofold. First, all appropriate scenarios are considered to evaluate the operational risk of the solution. The introduction of biobjective, that is, operational risk and delay cost, can provide a more efficient solution with appropriate weights and thus eliminate highly conservative solution. Second, without using capacity scenarios as constraints, the number of constraints in this model will not dramatically increase with the increasing number of scenarios. Therefore, we can efficiently avoid computationally intractable problem. Using real-world air traffic network data associated with simulated weather data, computation experiments demonstrate that our proposed model has generality that is suitable for solving SATNFO problem under different capacity scenarios. Moreover, the number of constraints is far less than stochastic model with nonanticipative constraints, which means the presented model has less computation complexity. Besides, we provide an appropriate range of weight coefficients to get a better tradeoff between operational risk and flight delay cost in practical ATFM decision-making.

The remainder of the paper is organized as follows.  Section 2 presents the mathematical model of our SATNFO problem, including the definition of operational risk.  Section 3 presents computation experiments that evaluate the efficacy of our SATNFO model, that is, the biobjective 0-1 integer programming model.  Section 4 gives conclusion and future research.

## 2. Mathematical Model of SATNFO

In this section, our mathematical model of SATNFO problem is introduced in detail. Firstly, the operational risk is defined via probabilistic risk assessment method to evaluate the effect of capacity uncertainties on ATFM. And then, full mathematical formulation of SATNFO problem is presented.

Before the introduction of mathematical model of SATNFO problem, two concepts should be indicated here: (1) air traffic network: airspace is divided into sectors. Each sector or airport can be represented as a node in the network. If a flight is planned to fly from one node to another, there will be a directed arc between these two nodes; (2) capacity: for each sector or airport, there are a limited number of flights that can fly within it in every time unit. It can be easily influenced by convective weather.

### 2.1. Definition of Operational Risk

During the modeling, we introduce the concept of operational risk into SATNFO problem to deal with the uncertainties. Generally, operational risk is defined [31] as the risk of a change in value caused by the fact that actual losses, incurred for inadequate or failed internal processes, people, and systems, or from external events, differ from the expected losses. The operational risk in SATNFO problem can be defined as the extra flight delay cost caused by unpredictable capacity changes and inappropriate flight plan. A robust set of flight plans for all scheduled flights receives less impact resultant from capacity uncertainties and thus with lower operational risk.

In order to quantify the operational risk induced by capacity uncertainties, we adopt the probabilistic risk assessment (PRA) method [32–34]. PRA is a methodology to evaluate risks associated with a complex engineered technological entity. It has been successfully used in agrochemicals analysis [35], nuclear power industry [36], and many other complex engineering fields [37]. In PRA method, the risk is characterized by two elements: the magnitude of all possible adverse consequences (*I*_*i*_,  *i* = 1,…, *N*, suppose *N* possible adverse consequences in total) and the likelihood of each consequence (*P*_*i*_,  *i* = 1,…, *N*). And, the risk is defined as ∑_*i*=1_^*N*^ *I*_*i*_ · *P*_*i*_. As for SATNFO problem, the magnitude of adverse consequence is the extra flight delay cost caused by capacity uncertainties, and the likelihood is determined by the capacity statistical distribution. According to PRA method, the steps to quantify the operational risk in SATNFO problem are as follows.

#### 2.1.1. Capacity Statistical Distribution Modeling

In practical ATFM, the convective weather dynamics often lead to capacity uncertainties for the airports or airspace sectors in a network. A capacity statistical distribution represents possible values of capacity and its distribution and their changes with weather condition along time horizon. The modelling of capacity statistical distribution is built on the basis of the three assumptions and principles.

1. The airspace is divided into *M* regions (denoted by {*S*_1_, *S*_2_,…, *S*_*M*_}), and each region consists of several sectors and airports. The time period is divided into *Q* time stages, and each time stage consists of certain smallest time units. When changes of weather spread in the airspace, it can only influence one of the regions during a time stage. Thus, the spreading process can be equally expressed as the capacity of this region decreasing lasting a certain time units.
2. The extent of weather impact on capacity is divided into *N* degrees, and the corresponding weather modes are denoted by {*D*_1_, *D*_2_,…, *D*_*N*_}.
3. Let scenario tree [38] describe the capacity statistical distribution. The scenario tree may be various in different time period because of various weather conditions. One node on a scenario tree represents a certain weather mode in a certain region at a certain time stage, according to which we can calculate capacity and its probability of each region (i.e., a capacity distribution of the whole airspace). We call a node as a capacity scenario.

Based on above assumptions, the steps of modelling capacity statistical distribution can be described as follows.

**Figure 1:**
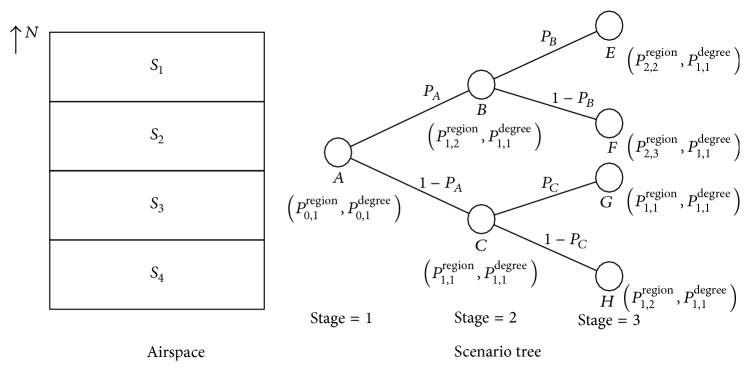
Demonstration of scenario tree.

(1) Get the initiative scenario, that is, a weather mode and a region it influenced in time stage 1. Let a scenario be denoted by (*P*_*k*,*l*_^region^, *P*_*i*,*j*_^degree^), where *P*_*k*,*l*_^region^ represents the probability of the weather spreading from *S*_*k*_ to *S*_*l*_, and *P*_*i*,*j*_^degree^ represents the probability of weather mode that transfers from *D*_*i*_ to *D*_*j*_. At the initial moment, if a weather mode whose degree is *D*_1_ influences the *S*_1_ region, we use (*P*_0,1_^region^, *P*_0,1_^degree^) to represent this scenario.
(2) Analyze the impact of weather along time horizon, including the weather spreading modes (decide which region will be influenced) and the degree of its impact. The impact of weather is uncertain. As time stage transfers from time stage *t* to *t* + 1, the changes of weather may spread to different regions with a certain probability. And moreover, for a region, the impact degree of weather is also uncertain. Hence, a scenario can generate several subsequent scenarios with a certain decision probability. And, along the time horizon, a scenario tree can be established. Suppose *Q* = 3, *N* = 1, airspace is divided into 4 regions (shown in the left figure of Figure 1), weather spreading from north to south, and a scenario tree can be established as the right figure of Figure 1. In this scenario tree, the initiative scenario, that is, the root node *A*, is (*P*_0,1_^region^, *P*_0,1_^degree^).
(3) Based on the process of establishing scenario tree, we can get a dynamic probabilistic capacity distribution and decision probability. For example, in Figure 1, the probability of scenario *G* happening is
  (1) $$\begin{array}{c} P\left(G\right)=P\left(A\right)\cdot P\left(C\;|\;A\right)\cdot P\left(G\;|\;C\right). \end{array}$$
  Scenario *G* represents a capacity distribution of the whole airspace at time stage 3. According to its information (*P*_1,1_^region^, *P*_1,1_^degree^), we can calculate the capacity for each sector or airport. And the corresponding probability *P*(capacity_*it*_) is *P*(*G*). Furthermore, for each sector or airport, we can obtain its probability of its possible capacity at time stage *t* according to all possible scenarios that may appear in stage *t*.

#### 2.1.2. Extra Delay Cost Calculation

Extra flight delay cost is the magnitude of adverse consequence caused by capacity uncertainties. Flight delay may be caused if capacity decreases; that is, the extra delay cost resulting from capacity decreases. The number of flights flying in the sector or airport may exceed capacity which is decreased due to the dynamics of convective weather. Hence, some flights should execute airborne holding or ground holding, which leads to extra flight delays. And, the extra flight delay is regarded as the time for a flight to cross the sector with minimum velocity. To simplify the calculation we assume that there is no more flight coming during this waiting period, which means we take no account of the delay propagation in this model.

When the traffic flow in sector *i* at time *t* is *N*_*it*_, extra airborne holding time AED_*it*_ caused by the capacity decreases in sector *i* at a certain time *t* can be expressed as below:

(2) $$\begin{array}{c} AED_{it}=\left(N_{it}-Cs_{it}\right)\cdot Pass_{i} \end{array}$$

where Cs_*it*_ is the capacity of sector *i* at time *t* and Pass_*j*_ is the time that a flight needed to cross sector *i* with minimum velocity.

Moreover, if the flow exceed the arriving capacity of airport *a*, it can also cause extra airborne holding:

(3) $$\begin{array}{c} AED_{at}=N_{at}-Ca_{at}. \end{array}$$

Ca_*at*_ is the arriving capacity of airport *a* at time *t*. Similarly, if the flow exceeds the departure capacity of airport *d*, the extra ground-holding delay cost GAD_*dt*_ can be expressed as:

(4) $$\begin{array}{c} GED_{dt}=N_{dt}-Cd_{dt}. \end{array}$$

Cd_*dt*_ is the departure capacity of airport *d* at time *t*.

#### 2.1.3. Operational Risk Formulation

According to PRA method, the operational risk of SATNFO problem can be defined as below:

(5) $$\begin{array}{c} Operational\;Risk=\sum extra\;delay\times p\left(capacity_{it}\right). \end{array}$$

It can also be written as

(6) $$\begin{array}{c} Operational\;Risk=\;Risk_{airborne}\;+Risk_{ground}. \end{array}$$

Risk_airborne_ is the risk from airborne holding and can be represented as below:

(7) Riskairborne=∑t,iNit−Csit·Passi·PCsit+∑t,aNit−Caat·PCaat,

where *P*(Cs_*it*_) is the probability that the capacity of sector *i* at time *t* is Cs_*it*_.

Risk_ground_ is the risk from ground holding that can be represented:

(8) $$\begin{array}{c} Risk_{ground}=\underset{t,d}{\sum }\left(N_{dt}-Cd_{dt}\right)\cdot P\left(Cd_{dt}\right), \end{array}$$

where Cd_*dt*_ is the departure capacity of airport *d* at time *t*.

Since the cost of airborne holding is much greater than ground holding, it is necessary to be considered in the definition of the operational risk. Hence, the total risk function should be improved as

(9) Operational  Risk=wairborne·Riskairborne+wground·Riskground,

where *w*_airborne_ > *w*_ground_.

### 2.2. Mathematical Formulation of SATNFO Problem

In our SATNFO model, the minimization of operational risk, which evaluates the effect of capacity uncertainties on ATFM, is introduced as an objective. And, the minimization cost of total flight delays is the other objective.

The air traffic network in our SATNFO model is based on deterministic ATFMO model. To our knowledge, the 0-1 integer programming model [9] proposed by Bertsimas et al. (BLO model) has high computational efficiency and also accuracy to depict the real ATFM actions such as ground holding, airborne holding, speed control, and rerouting. Thus, we build our SATNFO model based on BLO model.

#### 2.2.1. Decision Variable

We use the same definition of decision variable in BLO model:

(10) $$\begin{array}{c} z_{i,t}^{f}=\left\{\begin{array}{cc} 1 & if\;flight\;f\;arrive\;sector\;i\;by\;time\;t\\ 0 & otherwise. \end{array}\right. \end{array}$$

Note that we use “by” instead of “at.” This definition means once the decision variable is 1 at time unit *t*, the value of decision variables in the subsequent time units must be 1. It could also be applied to airports. If flight *f* takes off from airport *i* by time *t*, the decision variable is 1 (similarly, if flight *f* lands at airport *j* by time *t*, the decision variable is 1).

#### 2.2.2. Notations

The notations in the SATFNO model which are similar to BLO model are listed as follows:

 
*K*: set of sectors,
 
*K* ^*f*^: set of sectors that could be flown by fight *f*,
 
*P*: set of airports,
 
*P* ^*f*^: set of airports that could be flown by fight *f*,
 
*T*: set of time units,
 
*F*: set of flights,
 
*A*_*i*_^*f*^: sets of sector *i*′s ancestor sectors for flight *f*,
 
*S*_*i*_^*f*^: sets of sector *i*′ subsequent sectors for flight *f*,
 Cid_*p*_(*t*): ideal planning departure capacity of airport *p* at time *t*,
 Cia_*p*_(*t*): ideal planning arrival capacity of airport *p* at time *t*,
 Cis_*i*_(*t*): ideal planning capacity of sector *i* at time *t*,
 dep_*f*_: departure airport of flight *f*,
 arr_*f*_: arrival airport of flight *f*,
 Pass_*fij*_: minimum number of time units for air crafts to pass through sector *i*,
 
$T_{f}^{i}[\underline{T_{f}^{i}},\bar{T_{f}^{i}}]$: time window for flight *f* to arriving $\bar{T_{f}^{i}}$: the maximum time units for flight *f* to arrive at sector *i* (depart or arrive at airport *i*),
 
$\underline{T_{f}^{i}}$: the minimum time units for flight *f* to arrive at sector *i* (depart or arrive at airport *i*),
 
$\bar{T_{f}^{i}}$: the maximum time units for flight *f* to arrive at sector *i* (depart or arrive at airport *i*).

#### 2.2.3. Objective Functions

In SATNFO model, we formulate a weighted biobjective 0-1 integer programming model. The objective consists of two parts: the operational risk and total flight delay cost. Thus, the objective function is as below:

(11) $$\begin{array}{c} objective\;function=\left(1-\beta \right)\cdot delay\;cost+\beta \cdot Risk_{total}. \end{array}$$

Note that the deterministic model is a particular case of this model when *β* = 0.

*(1) Delay Cost*. We refer to the definition in BLO model and classify the delay into two different parts: airborne holding AH and ground holding GH:

(12) AH=∑f∈F ∑t∈Tfarrft−Tfarrf_·zarrf,tf−zarrf,t−1f−∑t∈Tfdepft−Tfdepf_·zdepf,tf−zdepf,t−1f

(13) $$\begin{array}{c} GH=\underset{f\in F}{\sum }\;\underset{t\in T_{f}^{dep_{f}}}{\sum }\left(t-\underline{T_{f}^{dep_{f}}}\right)\cdot \left(z_{dep_{f},t}^{f}-z_{dep_{f},t-1}^{f}\right). \end{array}$$

We calculate them separately and assign airborne holding a higher coefficient as it costs more resources than the other one. Then, we add up airborne holding and grounding holding to get total delay cost, which is

(14) $$\begin{array}{c} delay\;cost=\alpha_{ah}\cdot AH+\alpha_{gh}\cdot GH. \end{array}$$

*(2) Operational Risk.* According to our definition in the previous section, the operational risk is extra delay cost caused by unpredictable capacity changes. We have obtained operational risk through (1)–(9).

#### 2.2.4. Constraints

Consider

(15) $$\begin{array}{c} \underset{f\in F:dep_{f}=p}{\sum }\left(z_{dep_{f},t}^{f}-z_{dep_{f},t-1}^{f}\right)\leq Cid_{p}\left(t\right)\;\forall p\in P,\;\;t\in T \end{array}$$

(16) $$\begin{array}{c} \underset{f\in F:arr_{f}=p}{\sum }\left(z_{arr_{f},t}^{f}-z_{arr_{f},t-1}^{f}\right)\leq Cia_{p}\left(t\right)\;\forall p\in P,\;\;t\in T \end{array}$$

(17) ∑i∈Kf:f∈Fmax0,zi,tf−∑i′∈Sifzi′,tf≤Cisit∀i∈K, t∈T

(18) $$\begin{array}{c} z_{i,t-1}^{f}-z_{i,t}^{f}\leq 0\;\forall t\in T,\;\;f\in F,\;\;i\in K_{i} \end{array}$$

(19) $$\begin{array}{c} z_{i,t-1}^{f}\leq \underset{i^{\prime }\in A_{i}^{f}}{\sum }z_{i^{\prime },t-pass_{fij}}^{f}\;\forall t\in T,\;\;f\in F,\;\;i\in K_{i} \end{array}$$

(20) $$\begin{array}{c} z_{i,\bar{T_{f}^{i}}}^{f}\leq \underset{i^{\prime }\in S_{i}^{f}}{\sum }z_{i^{\prime },\bar{T_{f}^{i^{\prime }}}}^{f}\;\forall f\in F,\;\;i\in K^{f} \end{array}$$

(21) $$\begin{array}{c} \underset{i^{\prime }\in S_{i}^{f}}{\sum }z_{i^{\prime },\bar{T_{f}^{i^{\prime }}}}\leq 1\;\forall f\in F,\;\;i\in K^{f} \end{array}$$

(22) $$\begin{array}{c} z_{i,t}^{f}\leq 0\;\forall f\in F,\;\;i\in K^{f}⋂P^{f},\;\;t\in T:t<{\underline{T}}_{f}^{i} \end{array}$$

(23) $$\begin{array}{c} z_{i,t}^{f}-z_{i,t-1}^{f}=0\;\forall f\in F,\;\;i\in K^{f}⋂P^{f},\;\;t\in T:t<{\bar{T}}_{f}^{i} \end{array}$$

(24) $$\begin{array}{c} z_{i,t}^{f}\in 0,1\;\forall f\in F,\;\;i\in K^{f}⋂P^{f},\;\;t\in T_{f}^{i}. \end{array}$$

Constraints (15) and constraints (16) give the limitation of departure and arrival capacity of airport *p* at time *t*. They ensure that the number of flights which take off from airport *p* will not exceed the departure capacity of airport *p* at time *t* and that the number of flights which land on airport *p* will not exceed the arrival capacity of airport *p* at time *t*. Constraints (17) stipulate that the number of flights that arrive at sector *k* will not exceed the capacity of sector *k* at time *t*. Closely related to the definition of decision “by,” constraints (18) guarantee the time continuity of every flight. Constraints (19) state that if flight *f* arrives at sector *i* at time *t*, it must have been arrived at its ancestor node before time *t* − pass_*fij*_. Constraints (20) state that if flight arrives at sector *i* it will arrive at its subsequent sectors. Constraints (21) present that, for every flight that has arrived at sector *k*, it can only arrive at one of its subsequent sectors. Constraint (22) and Constraint (23) present the time-window limitation for every flight and the sectors a flight will pass by. Constraints (22) guarantee that the flight *f* cannot arrive at sector *i* before the minimum time we planned. Constraints (23) ensure that flight *f* should arrive at sector *i* before the maximum time we planned or the flight will never arrive at sector *i*. Finally, constraints (24) state that decision variables are Boolean values which make our model a 0-1 programming model.

## 3. Experiments

In this section, two sets of experiments are carried out to evaluate the efficacy of our SATNFO model, that is, the biobjective 0-1 integer programming model. In the first set, we analyze the effect of parameter setting, that is, the weight coefficient of risk (*β*) in our SATNFO model, and thereby provide an appropriate range of weight coefficient to get a better tradeoff between operational risk and flight delay cost in practical ATFM decision-making. In the second set, we show the generality of our mathematical model by solving a SATNFO problem under various scenarios. After that, the computational complexity of our SATNFO model is analyzed.

As for the experimental data, we extract a real air traffic network from the northern airspace of China, which consists of 25 sectors. A time horizon of two hours and 12 minutes is considered, and it is divided into 22 unites, 6 minutes each. Two sets of flight plans, consisting of 55 flights and 220 flights, respectively, are considered to be optimized for different purposes, which will be explained later. Capacity instances are generated based on simulated weather data, and the corresponding generation method will be shown in Section 3.1. All experiments are performed using optimization programming language with CPLEX 12.5.1, on a PC with Inter core i5-3470 processor, 3.20 GHz, and 12 GB RAM Microsoft Windows 7 OS.

In the following of this section, the details of capacity instances generation, two sets of experiments, and computational complexity of our SATNFO model are presented in order.

### 3.1. Capacity Instances Generation

As mentioned previously, a scenario tree represents possible values of capacity and its distribution and their changes with weather condition along time horizon. In this subsection, we generate 6 different capacity instances, that is, 6 different scenario trees based on simulated weather data. The steps of capacity instances are described as follows.

**Figure 2:**
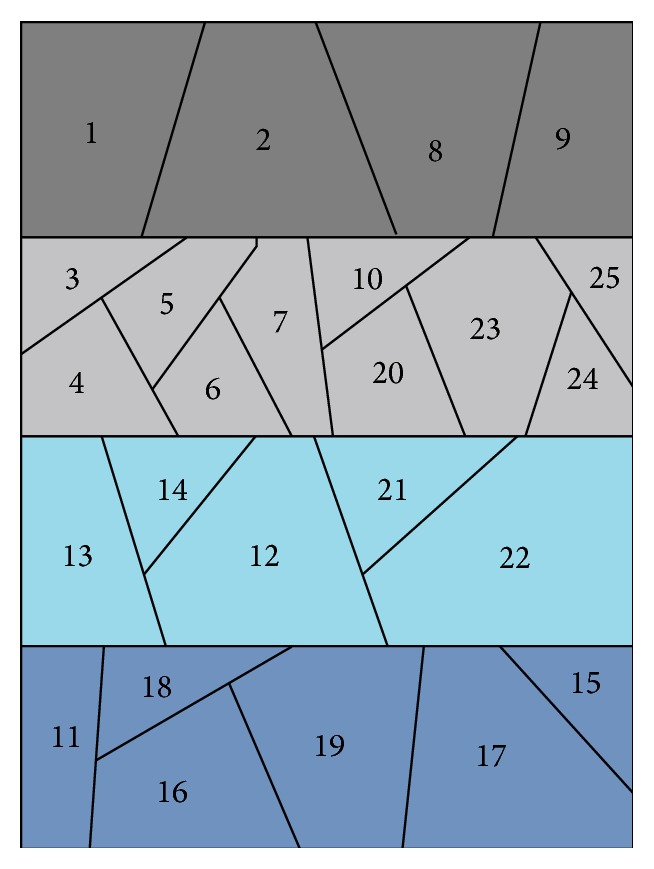
A schematic diagram of dividing airspace into 4 regions.

*(1) Partition of Airspace.* The airspace consisting of 25 sectors is divided into 4 regions.  Figure 2 is a schematic diagram of the airspace partition, thick lines are boundary of each region, and the value in sector denotes sector number. Weather has the same impact on sectors which are in a same region. Each region contains a set of sectors as follows:

 
*S*_1_: 8, 9, 1, 2;
 
*S*_2_: 3, 4, 5, 6, 7, 10, 20, 23, 24, 25;
 
*S*_3_: 13, 14, 12, 21, 22;
 
*S*_4_: 11, 16, 18, 19, 17, 15.

*(2) Definition of Impact of Weather on Capacity.* We suppose that the degree of impact of weather on capacity is divided into three classes, represented by ratio between current capacity under changes of weather and the ideal capacity which is a predicted and deterministic value. Three ratio values are denoted by {*A*, *B*, *C*}. The values of {*A*, *B*, *C*} are influenced by weather conditions. In our simulated weather data, there are two sets of them: DEG1 62.5%, 50%, and 37.5% and DEG2 50%, 37.5%, and 25%.

*(3) Definition of Weather Spreading Modes.* In our simulated weather data, there are three weather spreading modes for describing changes of weather.

 
*MODE 1: North-to-South Spreading.* Initially, changes of weather influence *S*_1_ with a certain degree *m*  (*m* ∈ *A*, *B*, *C*), and its probability is *P*_*m*_. In the following period, extreme weather leaves *S*_1_, so the capacity in *S*_1_ recovers. At the same time, changes of weather influence *S*_2_ and the degree of capacity decrease transfers from *m* to degree *n*  (*n* ∈ *A*, *B*, *C*). The transfer probability can be expressed as *P*_*mn*_. According to this rule, the changes of weather transfer to *S*_3_, *S*_4_ sequentially.
 
*MODE 2: Expanding from Center.* In this mode, changes of weather influence *S*_2_ first. In the next time period, changes of weather may spread to the north *S*_1_ or spread to the south *S*_3_. Then, if the change of weather is in *S*_3_, it would spread to *S*_4_. Otherwise, the changes of weather would leave this airspace and all capacities recover straight after. The definition of transfer probability is similar to mode 1.
 
*MODE 3: South-to-North Spreading.* In this mode, changes of weather influence *S*_4_ first and then spread to *S*_3_, *S*_2_, and *S*_1_ sequentially. The definition of transfer probability is similar to mode 1 and mode 2.

*(4) Capacity Instances.* Having gotten above three spreading modes and two sets of impact degrees of weather on capacity, we can get 6 capacity instances, denoted by the following:

 Instance 11 (MODE1, DEG1), Instance 12 (MODE1, DEG2),
 Instance 21 (MODE2, DEG1), Instance 22 (MODE2, DEG2),
 Instance 31 (MODE3, DEG1), Instance 32 (MODE3, DEG2).

According to each instance, we can generate its corresponding scenario tree based on the given weather data.

### 3.2. Risk Weight Coefficient Analysis

In this experiment, we analyze the effect of parameter setting, that is, the weight coefficient of risk *β*, in our SATNFO model. Hence, the relationship between *β* and total delay and the relationship between *β* and operational risk are analyzed concretely. For this experiment, a set of flight plans which consists of 55 flights is optimized under capacity instance “Instance 21.”

**Figure 3:**
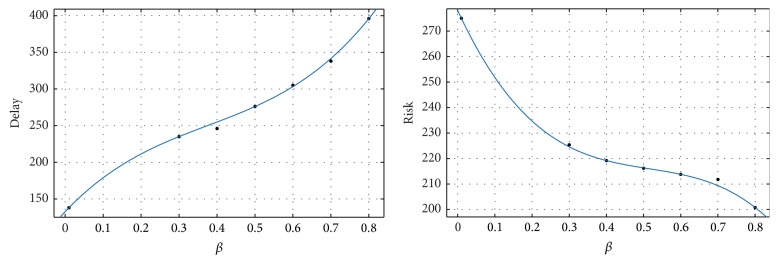
Demonstration of delay cost and risk with *β*.

Table 1 shows corresponding delay and risk with various *β*.  Figure 3 shows the relationship between *β* and delay (left) and the relationship between *β* and risk (right).

From Figure 3, we find that operational risk decreases and total delay increases with the increase of *β*. It means that when we add risk as one objective of SATNFO problem, the optimized flight plans are relatively conservative, that is, allowing more delays for safety operation. Further, the optimized flight plans become more conservative with the increase of risk weight coefficient. Thus, decision-makers can choose their appropriate risk coefficient to optimize flight plans for all scheduled flights using our SATNFO model. According to their experience and the real condition, they can obtain an acceptable tradeoff between optimization and robustness.

More important, the analysis of Figure 3 can provide information that helps decision-makers to give an appropriate value of *β* and get a better tradeoff. In the left figure, we can find that the delay cost increases almost linearly with the increase of *β*. In the right figure, the pace of risk decreasing with *β* is fast at first and then slows down. When *β* is less than 0.4, the risk will decrease obviously while the increase of delay cost is relatively slow; when *β* is more than 0.4, the risk decreases become slow while the increase of delay cost is dramatic. Therefore, adjusting *β* below 0.4 will help us to gain a small risk with a relatively lower delay cost.

### 3.3. Generality Analysis of Our SATNFO Model

In this experiment, we use our 6 generated capacity instances to test the generality of our SATNFO model. Moreover, we have increased the number of flights to 220. The value of *β* is set to 0.4.

**Figure 4:**
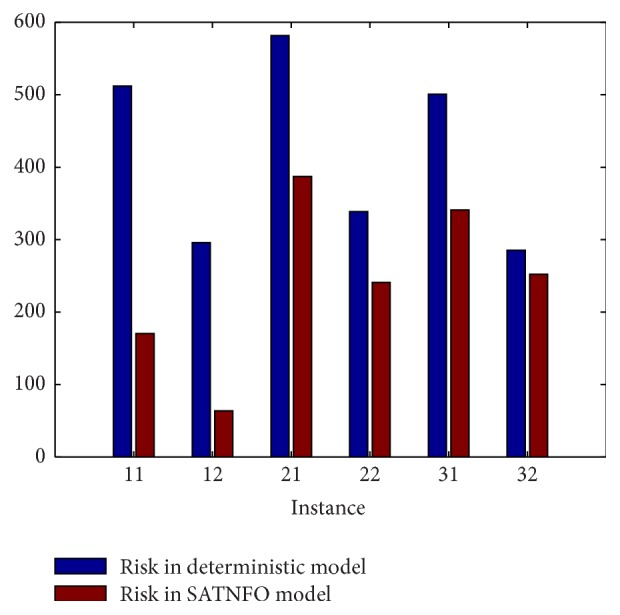
Demonstration of risk in deterministic model and our SATNFO model.

In each instance, capacity distribution changes according to its weather spreading mode and impact degree of weather on capacity.  Table 2 illustrates the risk in deterministic model and our SATNFO model. The first column represents different capacity instances. The second and third columns list corresponding delay and operational risk obtained by our SATNFO model for each capacity instance. The last column is the operational risk if we do not consider changes of weather (the optimization result of the deterministic model).  Figure 4 shows the comparison between the operational risk under deterministic model and our SATNFO model. In all 6 different capacity instances, our SATNFO model can obtain flight plans with less risk.

Note that our model can efficiently reduce operational risk under different capacity instances. Moreover, it can demonstrate the superiority of our SATNFO model on aspect of generality, since we can solve SATNFO problem efficiently with increased number of flights (from 55 flights to 220 flights), no matter what kind the capacity uncertainty is.

Furthermore, we find out that the results we obtain reflect the real situation. This also illustrates the rationality of our proposed model. In our capacity instances, south part of this area refers to Shanghai and its surrounding areas; the center part refers to Beijing (the capital of China) and its surrounding areas; and north part of this area refers to Inner Mongolia. The airline operation is far busier in Beijing and Shanghai than that in Inner Mongolia. So it is reasonable that capacity distribution changes which start from center and south (Instance 21, Instance 22, Instance 31, and Instance 32) will lead to more risk than those which start from north (Instance 11, Instance 12). Our experiment exactly shows this difference. From this perspective, our SATNFO model is consistent with the reality, which illustrates its rationality.

### 3.4. Computational Complexity Analysis

If the numbers of sectors, flights, and time units are denoted by *S*, *F*, and *T*, respectively, then the dimension of decision variable is |*T* | ·|*F* | ·|*S*|. The upper bound of constraints is 2 × |*F* | ×|*T* | ×|*F* | ×|*S* | +2 × |*F* | ×|*T*|.

Table 3 shows the numbers of constraints in our proposed SATNFO model and in stochastic-programming with nonanticipative constraints (denoted by SPNAC model hereafter) [26]. From the table, we found that to solve a SATNFO problem with the same scale, the constraints in our proposed model are far more less than in the SPNAC model.

## 4. Conclusion and Future Research

In this paper, we successfully provide a novel weighted biobjective 0-1 integer programming model to deal with the uncertainty in SATNFO problem. In this model, two objectives are the minimization of the cost of total flight delays and the minimization of the operational risk. In order to evaluate the effect of capacity uncertainties on ATFM, the operational risk is specifically defined via probabilistic risk assessment method. By assigning different weight coefficients on risk and delay cost, some efficiency is compromised to make our flight schedule more robust. This model allows decision-makers to adjust risk weight coefficients, so that they can make tradeoff between risk and delay costs. Moreover, because we introduce the operational risk function into objective instead of using capacity constraints under uncertainties, we avoid the dramatic increase of the number of constraints and thus avoid suffering the computational intractability.

Through computation experiment, we find out an appropriate range of risk weight coefficient that enables decision-makers to gain a small risk with a relatively lower delay cost. Successful tests under different capacity distribution instances confirm the generality of our SATNFO model. Moreover, result shows the number of constraints is far less than nonanticipative constraints in stochastic programming, which illustrates the high computational quality of this model.

Our model could be further improved in the future. First, various kinds of uncertainty sources could be considered besides capacity changes. For example, the flight demand is also an uncertain element in air traffic flow management problem. Taking various kinds of uncertainties can make our model closer to the real-world application. Second, the choice between risk and delay cost is totally decided by setting risk weight, which is an artificial decision in this model. Although it is flexible, to some extent, the unreliability is inevitable. It is possible to introduce a feedback mechanism in future studies that the risk weight could be automatically adjusted according to the risk value during the optimization. In this case, we can avoid the unreasonable decision and make our model more reliable.

## Figures and Tables

**Table 1:** β versus delay and risk.

β	Delay	Risk
0.01	138	275
0.3	235	225.4
0.4	246	219.2
0.5	276	216.2
0.6	305	213.8
0.7	338	211.8
0.8	396	200.8

**Table 2:** Delay, risk in SATNFO model, and risk in deterministic model.

Instance	Delay-uncertain	Risk-uncertain	Risk-determine
Instance 11	2260	170.3	512.0
Instance 12	2151	63.6	296.0
Instance 21	2415	387.2	581.7
Instance 22	2318	240.8	338.7
Instance 31	2454	340.8	500.8
Instance 32	2365	252.3	285.4

**Table 3:** Numbers of constraints of SATNFO model and SPNAC model.

Model	Flight	Time stage	Sector	Degradation degree	Constraints
SPNAC	55	4	25	3	494971
SATNFO	55	4	25	3	42374
SATNFO	220	4	25	3	165530
